# Selenium Species: Uptake, Transformation and Toxicities in Differing Substrates and Their Pharmaceutical Applications

**DOI:** 10.1155/MBD.1994.265

**Published:** 1994

**Authors:** Katherine J. Kaye

**Affiliations:** 1 School of Geography Mansfield Road Oxford OX1 3TB United Kingdom


### SELENIUM SPECIES: UPTAKE, TRANSFORMATION AND TOXICITIES IN DIFFERING SUBSTRATES AND THEIR PHARMACEUTICAL APPLICATIONS

Katherine J. Kaye

School of Geography, Mansfield Road, Oxford OX1 3TB, U.K.

### I. Introduction

### A. Characteristics of Se

Selenium is a member of the oxygen group of elements; it is primarily anionic but may form cations in non-aqueous solvents. Its normal oxidation states are selenate, the $6^+$ form (Se (VI); selenite, the $4^+$ (Se (IV); the very transient $2^+$ form, and the selenide $2^-$ form, to which it is easily reduced. Pulse radiolysis studies (e.g., Kläning and Sehested, 1986) reveal the presence of Se (V) forms in aqueous selenate/selenites, reflecting the rapid reduction or oxidation of the two higher forms. The structures of Se clusters have been investigated by Hohl, *et al.*, (1987). Selenium is an essential micronutrient; its biochemical functions have been reviewed briefly by Wendel (1992), and by Burk (1991) and Néve (1991) amongst others. It reduces the toxicities of Hg, Pb, Cd, Cu, Ni, and As (Cuvinaralar and Furness, 1991; Parizek, 1990; Paulsson and Lundbergh, 1991; Taketani, *et al.*, 1991; Ingersoll, *et al.*, 1990; Högberg and Alexander, 1986) and the nephrotoxicity of cisplatin (Baldew, *et al.*, 1991). By contrast, Se deficiency enhances the nephrotoxicity of cephaloridine, a betalactam antibiotic (Kays, *et al.*, 1991). It is strongly nucleophilic in biological systems and usually more reactive in biological molecules than its sulfur analogues (see, for example, Noszál, *et al.*, 1991).

**Table 1**

| *concentration acyl* | *concentration amine (M)* | $k^{obs}$ $sec^{-1}$ | *temperature* $^{o}C$ |
|---|---|---|---|
| N,Se-dibenzoylselenocysteine: | | | |
| $8.76 \times 10^{-5}$ | $3.38 \times 10^{-1}$ | $2.73 \times 10^{-3}$ | 29.8 |
| N,S-dibenzoylcysteamine: | | | |
| $8.76 \times 10^{-5}$ | $3.38 \times 10^{-1}$ | $2.31 \times 10^{-5}$ | 29.8 |

In this reaction (Table 1) with *n-butylamine* the selenocysteamine shows higher reaction rates by two orders of magnitude (Mautner, 1956); the same relationship holds for Se nucleophilicity over sulfur in chloroethyl sulfides and selenides (Kang and Spears, 1990). The selenocysteinyl sites of glutathione peroxidase (GSHPx) demonstrate similarly greater formation constants with methyl mercury than the corresponding thiol complexes which were thought to detoxify this form of Hg (Arnold *et al.,* 1986). Selenium radicals also react with amino acids by several orders of magnitude more rapidly than halide or pseudo-halide radicals (Badiello, 1992).

B. Approaches in research

For the last forty years, interest in selenium as a drug component has fluctuated, depending on several factors including the degree to which its chemistry was understood to differ from that of sulphur, the recognition of its physiological roles, investigations into its mutagenic capacities, and the degree to which scientists in one discipline were aware of advances made in other fields. Studies conducted on how organisms in seleniferous environments detoxified Se (Abu-Erreish, *et al.,* 1968; Fleming and Alexander, 1975; Chau, *et al*., 1976;) led to studies on methylation which in turn have led to the work by Ganther's group on selenobetaine (see below). The main commercial non-food use of Se has been in topical applications to counteract dandruff but its future lies in its anti-tumour and anti-toxic properties.

Selenium work has grown from two general approaches: first, observations from ovine, bovine and human models show significant correlations between Se levels and physiological parameters. Secondly, theory-based studies, often within the field of synthetic inorganic chemistry, exploit the characteristics which place Se in Group VIA of the periodic table, with O, S, Te and Po. Both approaches tend to exploit the notional analogies between S and Se: for example, metal-Se interactions in biology were first investigated as if they were metal-metallothionein interactions (e.g., Underwood, 1981; Tappel, 1980; Ganther, 1980); theory-based studies compare the known properties of S compounds with the activities of Se-substituted molecules, such as the substitution of selenium for chloride in 6-chloropurine ribosides (Mautner, 1956; Mautner, *et al.,* 1963; Scovill, 1992) or the synthesis of 2-chloro-ethylselenides (Khalil and Maslat, 1991). The use of Se as a ligand has been exploited more by synthetic inorganic chemists (e.g., Hoots, *et al.,* 1984) than by others in the field but its liganding characteristics are useful in the synthesis of biologically-active molecules.

C. Se metabolism

Se normally enters the body in its more oxidised forms, as selenate or selenite, is reduced to selenide, and is absorbed and/or excreted, depending on Se status, energy demands, dietary factors (e.g., lipid intake) and the toxic or heavy-metal burden carried by the organism. Selenite and selenious acid are toxic; its most oxidised state, selenate, is non-toxic. In normal metabolism, selenate reduction occurs via GSH to selenite which is then more quickly reduced by GSH reductase and/or methylation, as shown by the following equations (Table 2) , summarised in Whiting, *et al.,* (1980).

**Table 2**

(1) $HSeO_4^- + 2GSH \longrightarrow HSeO_3^- + GS\text{-}SG + H_2O$

(2) $HSeO^- + 4GSH \longrightarrow GS\text{-}Se\text{-}SG + GS\text{-}SG + OH^- + 2H_2O$

(3) $GS\text{-}Se\text{-}SG + GSH \longleftrightarrow GS\text{-}Se^- + GS\text{-}SG + H^+$

(4) $GS\text{-}Se\text{-}SG + NADPH \longrightarrow GS\text{-}Se^- + GSH + NADP^+$

glutathione reductase

(5) $GS\text{-}Se^- + H_2O \longleftrightarrow Se^o + GSH + OH^-$

(6) $GS\text{-}Se^- + NADPH + H_2O \longrightarrow HSe^- + GSH + NADP^+ + OH^-$

glutathione reductase

(7) $GS\text{-}Se^- + GSH \longleftrightarrow HSe^- + GS\text{-}SG$

(8) $HSe^- + [O] \longrightarrow Se^o + OH^-$

(9) $HSe^- + [O] + 2GSH \longrightarrow HSe^- + GS\text{-}SG + H_2O$

Selenate reduction by GSH and other sulfhydryl compounds is shown in Eq. 1; selenite reduction (Eq. 2) and further reactions take place in seconds. Reduction of selenite proceeds via the selenotrisulfide (GS-Se-SG) which is relatively stable , and via the persulfide, ($GS\text{-}Se^-$) which is highly reactive. Selenide is protected against oxidation (Eq. 8) by the presence of excess sulfhydryls (Eq. 9).

Protein incorporation may follow, or the product may be excreted after the formation of di- or trimethylselenides. The formation of methylated products, and the route of excretion, depends on the organism, on the form of selenium in the exposure, on the amount of selenium in the dose, on the selenium status of the organism, and on the presence of interactive factors, such as the sex of the animal, its hormone status (especially pregnancy or lactation in females), and the heavy-metal body load (e.g., Janghorbani, *et al.,* 1991; Parizek, 1990). That these interactive factors have a bearing on the use of Se in pharmaceuticals may be obvious; it is also significant that the selenobetaine synthetics, in bypassing the hydrogen selenide pool, may bypass at least some of these metabolic interactions which can potentiate Se or interaction factor toxicities.

### II. Se in biological materials

### A. Selenocysteine

Selenium may be most familiar through knowledge of the consequences of nutritional deficits, or through its role as the catalytic moiety of glutathione peroxidase. These concepts are related in that Se deficiency lowers GSHPx levels and activity with severe consequences for the organism. Briefly put, Se in GSHPx appears as a selenocysteine molecule, probably located at the N-terminal ends of alpha helices in what Epp, *et al*., (1983) postulate as a rather rigid betaalpha-beta structure. The main reaction of the catalytic cycle shuttles between the selenolate and seleninic acid state of selenocysteine. Selenocysteine biosynthesis, starting from a serine moiety, is genetically directed via a TGA stop codon. Bock, *et al*., (1991) proposed that the evidence presented by them and by the Stadtman group at the National Institutes of Health (e.g., Stadtman, 1990; Politino, *et al.,* 1991) justified the recognition of Se-cys as the 21st amino acid, using U as the symbol in the one-letter amino acid code. This convention is now fairly widespread in the relevant literature.

B. Protein binding

A number of issues inform the questions surrounding Se chemistry and biochemistry, and thus its pharmacology: these include the formation of Se proteins, and the inorganic chemistry of selenium *in vivo.* It is commonly held that Se incorporation occurs as a result of non-specific processes involving random substitution of Se for S during protein synthesis. For selenocysteine, this is not the case, and the general chemistry of selenoprotein formation is still poorly understood. The locations and characteristics of Se-binding proteins continue to be defined as Se-responsive conditions are proposed or verified. Of the number of other selenoproteins reported in the literature, and since the publication of the genetic encoding of Se-cys production, some of them have been examined for the TGA codon ( e.g., Hsu and Eckhert, 1991; Pumford,*et al.,* 1992), which has not been found.

Microvascular damage induced by sucrose has been reported to respond to Se supplementation: investigating this, Hsu and Eckhert (1991) report that in kidney microvascular nuclei over 90% of the radioselenium activity found was associated with the nucleus, and that of the two 22kDa proteins, one was associated with the nonhistone chromosomal fraction and the other with the nuclear protein matrix. That of the matrix was 73% homologous with bovine pancreatic ribonuclease; the protein associated with the chromosomal fraction had no trace homologies but had an N-terminal block.

The literature is more specific about binding in the liver, where GSHPx synthesis occurs and where over 70% of human GSHPx is found. A protein found in mouse liver binding a (hepatotoxic) reactive metabolite of acetominophen (Pumford, *et al,* 1992) has been found to be 97% homologous with a non-selenocysteine selenium-binding protein of 56 kDa (Kang and Spears, 1990). It is not known if the acetominophen metabolite binds to a selenol or selenide moiety of the protein or to the cysteinyl sulfhydryl groups. The role of Se in decreasing acetominophen hepatotoxicity is not agreed. Pumford and co-workers suggest that it contains a number of highly nucleophilic sites which scavenge electrophiles not detoxified by glutathione.

It is curious, however, that as late as 1991, Bock's group were able to publish a statement that "it is still not known how selenite enters the cell and how it is reduced to selenide" (Bock, *et*

*al.,* 1991: 516). In 1983, Epp, *et al*., were willing to write in reference to GSHPx that 'traces of alimentary selenium provide the prosthetic group, a selenocysteine residue'. The 1978 reference by Forstrom and others which they cited is not categorical or definitive about how alimentary Se is converted to selenocysteine, whether the initial Se is inorganic or a component of - for example - selenomethionine, a common selenoprotein found in whole grains. Forstrom suggests that selenocysteine could be synthesised *in vivo* from selenomethionine via selenocystathionine (Forstrom *et al.*, 1978); or, in the presence of GSH and GSH reductase, a cysteine-synthase-like reaction (Olson and Palmer, 1976) could take place with hydrogen selenide. Later investigations (e.g., Whanger *et al.,* 1991) indicate that the plasma Se of rats, monkeys, sheep and humans was associated predominantly with selenoprotein P, and that the association of Se with albumin was dependent on the selenomethionine content of the diet.

C. Mutagenic and clastogenic activity of Se

The organo-Se compounds tested by Ganther's group give negative results in genotoxicity tests. Others investigating organoselenium (e.g., Kang and Spiers, 1990; Cotgreave, *et al.*, 1991; el-Bhayoumi, *et al.*, 1992); the 2-chloroethylselenides synthesised by Kang and Spears are more than two orders of magnitude higher in nucleophilicity than their sulphur analogues. Czyrski and Inglot (1991) demonstrate the mitogenicity of some organo-Se compounds; Khalil and Muslat (1990[1]) Chromosomal aberrations, undetectable by the [then available] cytogenic method may be induced by lower doses of Se than those administered by Norppa, *et al.*, (1980a-c), as they observed. Their tests were negative in bone marrow or primary spermatocytes of mice after i.p. injection of 0.8 mg/kg bd.wt.; on CH bone marrow, 0.3-6.0 mg/kg bd.wt. injections of sodium selenite induced SCE and aberrations at the 3-6 mg/kg dose range. Sirianni and Huang (1983), testing 0-64 µg/ml of $NaSeO_3^{2-}$, $NaSeO_4^{2-}$ and $HSe^{2-}$ with and without S9, found the lower oxidation states induced more SCE than selenate in the CH V79 cell line. Ray (1984), measuring SCE induced by doses of [$7.95 \times 10^{-6}$] M $NaSeO_3^{2-}$, found that reduced glutathione was required for conversion of selenite to its SCE-inducing form, suggesting that this conversion runs on the same pathway as normal RBC metabolism of sodium selenite. Anjaria and Madhvanath (1988) noted that the presence of glutathione enhanced the selenite-induced genotoxicity in the yeast BZ34. The rate of genetic damage rose and then fell over the period of their five-hour experiments.

Kramer and Ames (1988), Hung, et al., (1988) and Garberg, et al., (1988) have demonstrated that selenite genotoxicity is a function of oxidative damage. Kramer and Ames ascribe this to the reaction of intracellular thiols and selenite, producing peroxide and superoxide radicals; Garberg

[1] A minor corrigendum appeared in *Mutation Res.* (1991) 242,7: 293.

and co-workers found that DNA fragmentation was inhibited by cyanide and mercuric chloride. Hung and co-workers found a significant protective effect against oxidative single-strand breaks (SSB) at the lower concentrations of $10^{-5}$-$10^{-7}$ M $NaSeO_3^{2-}$, but a significant damaging effect at $10^{-4}$ M $NaSeO_3^{2-}$. Balansky (1991) uses his battery of tests results, including a two week challenge to rats and mice of 10 ppm $NaSeO_3^{2-}$ in the drinking water, to assign a rôle of comutagen and co-clastogen to selenite. Selenium is thus an oxidant and an anti-oxidant in biological systems, depending on its form, route of entry into metabolism, and its co-occurrence with other factors.

### III. Selenium activity in anti-tumour agents

### A. Henry Mautner: Yale

Early developments of Se antitumour agents took place in the late 1950's and early 1960's through Henry Mautner's work in the Department of Pharmacology at Yale. By using the development of S-analogues of naturally-occurring purine and pyramidine bases - e.g., 6-mercaptopurine, 6-methylmercaptopurine and 2-thiouracil - as paradigms, Mautner synthesised a number of compounds between 1956 and 1962: 6-Se-purine, 6-Me-Se-purine, 2-selenouracil, 2,4-diselenouracil, 2-selenothymine (Mautner, 1956); and later, selenoguanine, diselenothymine, and 5-methylcytosine (Mautner, *et al.*, 1963). 6-Se-purine was prepared by the addition of sodium hydrogen selenide to 6-chloropurine; a similar reaction adding selenourea produced a 92% yield. The selenouracils were synthesised using selenourea instead of thiourea in the then-standard uracil synthetic methods.

Hypoxanthine (oxypurine), mercaptopurine and the 6-selenpurine were methylated, producing hypsochromic shifts in UV absorption with increasing atomic number, and an increase in acidity of the compounds. The polarisability of the carbonyl, thiocarbonyl and selenocarbonyl bonds increases in passing up the periodic table.

Of these compounds, the selenopurine, tested on the growth of mouse lymphoma L-1210 cells, demonstrated a level of activity equivalent to that of 6-mercaptopurine, then in use as a clinical anti-leukaemic agent. In other mouse tumour studies the thiopurine was more active. On the other hand, the selenopurine was more anti-bacterial and had a greater ability to inhibit formate incorporation into purines than the sulphur analogue. 6-selenopurine was also found to have Cu-chelating properties equal to those of 8-hydroxyquinoline and exceeding those of hypoxanthine. In an effort to improve the performance and stability of the selenopurine, Mautner's group (Mautner, *et al.*, 1963) made selenoguanine as an analogue of 6-thioguanine and compared this activity against implanted L5178Y lymphoma in mice, in 6C3HED lymphosarcoma cell lines, and in mice bearing an ascites cell form of sarcoma 180. In the latter case the selenoguanine produced tumour inhibition over a wider dose range; in the former whilst both thioguanine and selenoguanine were inhibitory, selenoguanine was the least toxic of the two. Selenocytosine was compared with thiocytosine as an anti-tumour agent, but neither compound significantly prolonged the survival time of mice implanted with the L5178Y lymphoma.

Mautner also studied the effects of Se-substitution on the lipophilicity of phenobarbital, a 2-selenobarbiturate (Mautner and Clayton, 1959), and compared the transacylation rates of thioacyl and selenoacyl analogs in reaction with *n*-butylamine (Mautner and Günther, 1961). Se substitution of O in phenobarbital increased the drug's lipophilicity.From this structure, Günther, *et al.*, (1992) have developed model photosensitising merocyanine dyes with a *circa* 100-fold enhancement of photogeneration efficiency for singlet oxygen.

B. Howard Ganther, Wisconsin

Howard Ganther and his group have worked on Se metabolism for over thirty years; their earliest work concerned agricultural herbivore metabolism of Se. Herbivores rely on plants, and therefore soils, for their trace element nutrition, and the primary concerns of Se research were those of deficiency - manifested in cardiac myopathy and so-called 'white muscle disease' - and of excess, manifested in 'blind staggers' - neurological and skeletal muscle impairment - and sloughing of hooves and other keratinised tissues. The principal concern was the detoxifying of Se through methylation ( Nahapetian, *et al.*, 1983; McConnell and Portman, 1952; Obermeyer, *et al.*, 1971; Foster, *et al.*, 1986); the production of hydrogen selenide was considered to be the intoxifying step in Se toxicity. The forms of Se most productive of MeSe were investigated for many years; in the course of these it was noted (Foster, *et. al.*, 1986b) that trimethylselenium could not be formed from selenobetaine by decarboxylation.

Methyl compounds were long held to be biologically inactive. However, two further types of discoveries have led to the present state of selenobetaine cytotoxins. First, methylated species can be remobilised by demethylation, in soils (Oremland, *et al.*, 1990); *in vivo*, trimethylselenonium ion, *via* betaine:homocysteine methyltransferase, can be demethylated to form dimethyl selenide and methione (Goeger and Ganther 1993). Secondly, whilst in theory methylated species should be active through their increased lipophilicity, no evidence of this was found until 1989, when Ganther's group reported an observation that methyl-Se was found in mouse kidney, where they didn't expect it (Stadtman, 1990). They also reported a synergistic toxicity between arsenite and methyl-Se compounds (Kraus and Ganther, 1989). In normal tissue seven organo-Se compounds were shown to be toxic when administered with arsenite (Table 3) and this toxicity held true for tumour suppression in dimethylbenz(a)anthracene induced mammary tumours in rats (Ip and Ganther, 1990).

**Table 3**

Methylseleninic acid (2 mg Se/kg)

Dimethylselenoxide (2 mg Se/kg)

Trimethylselenonium chloride (3 mg Se/kg)

Selenobetaine (2 mg Se/kg)

Selenobetaine methyl ester (2, 1, and 0.5 mg Se/kg)

Se-methylselenocysteine (2 mg Se/kg)

Selenomethionine (2 mg Se/kg)

In a later study (Ip and Ganther, 1992), the arsenite addition was dropped, and paired S/Se compounds were tested for DMBA-induced tumour suppression (cysteamine/selenocystamine; sulfobetaine/selenobetaine; S-methylcysteine; Se-methylselenocysteine). In the sulfur series, only cysteamine and S-methylcysteamine produced anticancer activity, and the levels required for these responses were 500-750 times as high as those of the corresponding Se analogues.

### IV. Conclusions

The mechanisms of Se genotoxicity both for normal and tumour cells are not settled, but it is established that an inverse relationship exists between Se content of soils and water and the incidence of human tumours. Ganther's group (Wilson, *et al.,* 1992) take the view that DNA fragmentation resulting from selenite exposure occurs during reductive metabolism and not from an accumulation of the methylated by-product. The methylated compounds alter the long-term proliferative potential of cells via mechanisms distinct from those associated with cell injury and death by necrosis. Se supplementation can inhibit tumorigenesis in rats, depending on the dose of the carcinogen and dietary fat intake (Ip, 1981). Banner and others (1981) found that pre-treatment with sodium selenite did not reverse or inhibit the acute inhibition of RNA and DNA synthesis induced by methyl-azoxymethanol (MAM); they conclude that Se exerts its protective effect by some mechanism other than interference with carcinogen activation by microsomes. Balansky (1991, 1992) has published two papers which he feels show conclusively that both Se genotoxicity and its antitumour effects are a function of timing of dose and the presence or absence of possible comutagens. It is clear that the exploitation of the reactivity of selenium must be mediated by the properties of its carrier molecules, and by a better understanding of its metabolic chemistry. This is impossible without better communication between structural/analytical chemists and biological chemists of their respective investigations and their results.

### References:

Abu-Erreish, G.M., E.I. Whitehead and O.E. Olson (1968) Evolution of volatile selenium from soils. *Soil Science* 106: 415-420.

Anjaria, K.B. and U. Madhvanath (1988) 'Genotoxicity of selenite in diploid yeast'. *Mutation Res.* 204: 605-614.

Arnold, A.P., K.-S. Tan and D.L. Rabenstein (1986) Nuclear Magnetic Resonance Studies of the Solution Chemistry of Metal Complexes, 23: Complexation of Methylmercury by Selenohydryl-Containing Amino Acids and Related Molecules. *Inorg. Chem.*, 25: 2433-2437.

Badiello, R. (1992) Physical-chemical properties of selenium-containing inorganic radicals and their reactivity with amino acids and enzymes. *Phosphorus, Sulfur and Silicon* 67: 7-10.

Balansky, R.M. (1991) Comutagenic and coclastogenic effects of selenium *in vitro* and *in vivo. Mutation Res.* 263: 231-236.

Balansky, R.M. (1992) Effects of sodium selenite and caffeine on mutagenesis induced by *N*-methyl-*N*-nitrosourea, *N*-methyl-*N'*-nitro-*N*-nitrosoguanidine and aflatoxin $B_1$ in *S. typhimurium. Mutation Res.* 269: 307-317.

Baldew, G.S., J.G. Mol, F.J. de Kanter, B. von Baar, J.J. de Goeij and N.P. Vermeulen (1991) 'The mechanism of interaction between cisplastin and selenite.' *Biochem. Pharmacol.* 41,10: 1429-1437.

Banner, W.P., Q.H. Tan, and M.S. Zedeck (1981) Studies of the mechanism by which selenium (Se) inhibits tumor formation. *Proc. Am. Assoc. Cancer Res.* 22: A457.

Bansal, M.P., C. Ip and D. Medina (1991) Levels and $^{75}$Se-labelling of specific proteins as a consequence of dietary selenium concentration in mice and rats. *Proc. Soc. Exp. Biol. Med.* 196: 147-154.

Böck, A., K. Forchhammer, J. Heider, W. Leinfelder, G. Sawyers, B. Veprek and F. Zinoni (1991) Selenocysteine: the 21st amino acid. *Molecular Microbiol.* 5: 515-520.

Burk, R.F. (1991) 'Molecular biology of selenium with implications for its metabolism.' *FASEB J.* 5: 2274-2279.

Chau, Y.K., P.T.S. Wong, B.A. Silverberg, P.L. Luxon and G.A. Bengert (1976) Methylation of selenium in the aquatic environment. *Science* 192: 1130-1131.

Cotgreave, I.A., P. Moldeus, L. Engman and A. Halberg (1991) 'The correlation of the oxidation potentials of dibenzo[1,4]-dichalcogenines to their antioxidance capacity in biological systems undergoing free radical-induced lipid peroxidation'. *Biochem. Pharmacol.* 42,7: 1481-1485.

Cuvinaralar M.L.A. and R.W. Furness (1991) 'Mercury and selenium interaction: a review'. *Ecotoxicol. and Environ. Safety* 21,3: 348-364.

Czyrski, J.A. and A.D. Inglot (1991) 'Mitogenic activity of selenoorganic compounds in human blood peripheral leukocytes'. *Experientia* 47,1: 95-97.

el-Bhaymoumi, K., Y.H. Chae, P. Upadhyaya, C. Meschter, L.A. Cohen and B.S. Reddy (1992) ' Inhibition of 7,12-dimethylbez(a)anthracene-induced tumors and DNA adduct formation in the mammary glands of female Sprague-Dawley rats by the synthetic organoselenium compound 1,4-phenylbis(methylene)seleno-cyanate.' *Cancer Res.* 52.9: 2402-2407.

Epp, O., R. Ladenstein, A. Wendel (1983) The Refined Structure of the Selenoenzyme Glutathione Peroxidase at 0.2-nm Resolution. *Eur. J. Biochem.* 133: 51-69.

Fleming, R.W. and M. Alexander (1975) Dimethylselenide and dimethyltelluride formation by a strain of *Penicillium. Appl. Microbiol.* 24: 424-429.

Forstrom, J.W., J.J. Zakowski and A.L. Tappel (1978) Identification of the Catalytic Site of Rat Liver Glutathione Peroxidase as Selenocysteine. *Biochemistry* 17: 2639-2644.

Foster, S.J., R.J. Kraus and H.E. Ganther (1986) The metabolism of selenomethionine, Se-methylselenocysteine, their selenonium derivatives, and trimethylselenonium in the Rat. *Arch. Biochem. Biophys.* 251:77-86.

Ganther, H.E. (1980) Interactions of vitamin E and selenium with mercury and silver. *Ann. N.Y. Acad. Sci.* 355: 212-225.

Garberg, P., A. Ståhl, M. Warholm and J. Högberg (1988) 'Studies of the role of DNA fragmentation in selenium toxicity.' *Biochem. Pharmacol.* 37,18: 3401-3406.

Goeger, D.E. and H.E. Ganther (1993) Homocysteine-dependent demethylation of trimethylselenonium ion and selenobetaine with methionine formation. *Arch. Biochem. Biophys.* 302: 222-227.

Günther, W.H.H., R. Searle and F. Sieber (1992) Photosensitising merocyanine dyes based on selenobarbituric acid. *Phosphorus, Sulfur and Silicon* 67: 417-424.

Hase, T. and P. Kangas (1991) Selenium dioxide and selenium disulfide as Grignard electrophiles. *Phosphorus, Sulfur and Silicon* 62: 281-282.

Högberg, J. and J. Alexander (1986) 'Selenium' in L. Friberg, G.F. Nordberg and V. Vouk, eds. *Handbook on the Toxicology of Metals*, 2nd ed. (Vol. I). Elsevier B.V., Amsterdam.

Hohl, D., R.O. Jones, R. Car and M. Parrinello (1987) The structure of selenium clusters - $Se_3$-$Se_8$. *Phys. Chem. Lett.* 139: 540-545.

Hoots, J.E., D.A. Lesch and T.B. Rauchfuss (1984) Peracid oxidation of Inorganic Chalcogen Ligands in Transition Metal Complexes. *Inorg. Chem.* 23: 3130-3136.

Hsu, M.-H., and C.D. Eckhert (1991) Molecular localisation of 75Se binding in microvascular nuclei. *FASEB J.* 5,4: 1208.

Hung, S.J., D.M. Sousa and A.B. Weitberg (1988) 'The effects of selenium and vitamin C on oxygen radical-induced DNA strand breaks'. *J. Exp. Clin. Cancer Res.* 7,4: 267-271.

Ingersoll, C.G., F.J. Dwyer and T.W. May (1990) 'Toxicity of Inorganic and Organic Selenium to

*Daphnia magna* (Cladocera) and *Chironomus riarius* (Diptera).' *Environ. Toxicol. and Chem.* 9,9: 1171-1181.

Ip, C. (1981) Anticarcinogenc effect of selenium supplementation in dimethylbenz[a]anthracene-induced mammary tumorigenesis. *Proc. Am. Assoc. Cancer Res.* 22: A455.

Ip, C. and H.E. Ganther (1990) Activity of methylated forms of selenium in cancer prevention. *Cancer Res.* 50: 1206-1211.

Ip, C. and H.E. Ganther (1992) Comparison of selenium and sulfur analogs in cancer prevention. *Carcinogenesis* 13: 1167-1170.

Janghorbani, M., E. Roberts, T. Hazell and B. Ting (1991) Effect of chemical form of dietary selenium on muscle selenium turnover. *FASEB J.* 5,4: 1999.

Kang, S.I. and C.P. Spears (1990) Structure-activity studies on organoselenium alkylating agents. *J. Pharmaceutical Sci.* 79: 57-62.

Kays, S.E., W.A. Crowell and M.A. Johnson (1991) The effects of Cu and Se deficiency on cephaloridine nephrotoxicity in rats. *FASEB J.* 5,4:1236.

Khalil, A.M. and A.D. Maslat (1991) Mutagenic effects of two syspected anticarcinogenic organoselenium compounds on *Salmonella typhimurium*. *Toxicol. and Environ. Chem.* 33: 23-29.

Kläning, U.K. and K. Schested (1986) Seleleium (V). A pulse radiolysis study. *J. Phys. Chem.* 90: 5460-5464.

Kramer, G.F. and B.N. Ames (1988) 'Mechanisms of mutagenicity and toxicity of sodium selenite ($Na_2SeO_3$) in *Salmonella typhimurium*.' *Mutation Res.* 201:169-180.

Krantz, F.. (1994) Protein-binding properties of two antitumour Ru(III) complexes to apotransferrin and the potential of transferin-conjugates in anti-tumour chemotherapy. *Metal-Based Drugs*: this issue.

Kraus, R.J. and H.E. Ganther (1989) Synergistic toxicity between arsenic and methylated selenium compounds. *Biol. Trace. Elem. Res.* 20: 105-113.

Mautner, H.G and E.M. Clayton (1959) 2-Selenobarbiturates. Studies of Some Analogous Oxygen, Sulfur and Selenium Compounds. *J. Am. Chem. Soc.* 81: 6270-6273.

Mautner, H.G and W.H.H. Günther (1961) The relative reactivity of thioacyl and selenoacyl analogs. *J.Am. Chem. Soc.* 83: 3342-3343.

Mautner, H.G. (1956) The Synthesis and Properties of Some Selenopurines and Selenopyrimidines. *J. Am. Chem. Soc.* 78: 5292-5294.

Mautner, H.G., S.-H. Chu, J.J. Jaffe and A.C. Sartorelli (1963) The Synthesis and Antineoplastic Properties of Selenoguanine, Selenocystosine and Related Compounds. *J. Am. Chem. Soc.* 6:36-39.

McConnell, K.P. and D.W. Roth (1952) Respiratory excretion of selenium. *Proc. Soc. Exp. Biol. Med.* 123: 919-921.

Messori, L. (1994) Transferrin: from inorganic biochemistry to medicine. *Metal-Based Drugs*: this issue.

Nahapetian, A.T., M. Janghorbani and V.R. Young (1983) Urinary trimethylselenonium excretion by the rat: effect of level and source of Se-75. *J. Nutr.* 113: 401-411.

Néve, J. (1991) 'Physiological and nutritional importance of selenium'. *Experientia* 47: 187-193.

Norheim, G. and K. Moksnes (1985) 'Distribution and Elimination of Selenium and Glutathione Peroxidase (GSHPx) in Chickens after Supplementation with Sodium Selenite or Selenomethionine.' *Trace Elements in Man and Animals 5.* Commonwealth Agricultural Bureaux, Slough.

Norppa, H., T. Westermarck, M. Laasonen, L. Knuutila and S. Knuutila (1980) 'Chromosomal effects of sodium selenite in vivo'. *Hereditas* 93: 93-105.

Noszál, B., W. Guo and D.L. Rabenstein (1991) Rota-microspeciation of Serine, Cysteine and Selenocysteine. *J. Phys. Chem.* 95: 9609 - 9614.

Obermeyer, B.D., I.S. Palmer, O.E. Olson, and A.W. Halverson (1971) Toxicity of trimethylselenonium chloride in the rat with and without arsenite. *Toxicol. Appl. Pharmacol.* 20: 135-146.

Oremland, D.S., *et al.*, (1990) 'Measurement of in-situ rates of selenate removal by dissimilatory bacterial reduction in sediments.' *Environ. Sci. Technol.* 24: 1157-1164.

Parizek, J. (1990) 'Health Effects of Dietary Selenium'. *Food and Chem. Toxicol.* 28,11: 763-765.

Paulsson, K and K. Lundbergh (1991) 'Treatment of mercury-contaminated fish by selenium addition.' *Water, Air and Soil Poll.* 56: 833-841.

Politino, M., Z. Veres, L. Tsai and T.C. Stadtman (1991) Partial purification and characterization of selenotRNA biosynthetic enzyme(s). *FASEB J.* 5,4: 388.

Pumford, N.R., B.M. Martin and J.A. Hinson (1992) A Metabolite of Acetominophen Covalently Binds to the 56kDa Selenium Binding Protein. *Biochem. Biophys. Res. Commun.,* 12: 1348-1355.

Ray, J.H. (1984) 'Sister-chromatid exchange induction by sodium selenite: Reduced glutathione converts $Na_2SeO_3$ to its SCE-inducing form.' *Mutation Res.* 141: 49-53.

Reamer, D.C. and W.H. Zoller (1980) Selenium: Biomethylation products from soil and seewage sludge. *Science* 208: 500-502.

Scovill, J.P. (1992) A convenient method for the synthesis of 6-selenopurine ribosides. *Phosphorus, Sulfur and Silicon* 70: 1-2.

Sirianni, S.R. and C.C. Huang (1983) 'Induction of sister chromatid exchange by various selenium compounds in chinese hamster cells in the presence and absence of S9 mixture.'

*Cancer Lett.* 18: 109-116.

Stadtman, T.C. (1990) 'Selenium biochemistry'. *Annu. Rev. Biochem.*, 59: 111-127.

Taketani, S., H. Kohno, R. Tokunaga, T. Ishii and S. Bannai (1991) 'Selenium antagonizes the induction of human heme oxygenase by arsenite and cadmium ions.' *Biochem. Internat.* 20,3: 522-527.

Tappel, A.L. (1980) Vitamin E and selenium protection from *in vivo* lipid peroxidation. *Ann. N.Y. Acad. Sci.* 355: 18-29.

Underwood, E.J. (1981) "The incidence of trace element deficiency diseases". *Phil. Trans. R.* Soc Lond. B294, 1071: 3-8.

Wendel, A. (1992) Biochemical functions of Selenium. *Phosphorus, Sulfur and Silicon*, 67: 405-415.

Whanger, P.D., J.A. Butler and J.T. Deagan (1991) Determination of the distribution of selenium between selenoprotein P, glutathione peroxidase and albumin in plasma. *FASEB J.* 5,4: 1993.

Whiting, R.F., L. Wei, H.F. Stich (1980) Unscheduled DNA synthesis and chromosome aberrations induced by inorganic and organic selenium compounds in the presence of glutathione. *Mutation Res.* 78: 159-169.

Wilson, A.C., H.J. Thompson, P.J. Schedin, N.W. Gibson, H.E. Ganther (1992) Effect of methylated forms of selenium on cell viability and the induction of DNA strand breakage. *Biochem. Pharmacol.* 43: 1137-1141.

**Received: September 7, 1993**

